# The Tacrolimus Metabolism Rate and Dyslipidemia after Kidney Transplantation

**DOI:** 10.3390/jcm10143066

**Published:** 2021-07-11

**Authors:** Gerold Thölking, Christian Schulte, Ulrich Jehn, Katharina Schütte-Nütgen, Hermann Pavenstädt, Barbara Suwelack, Stefan Reuter

**Affiliations:** 1 Department of Internal Medicine and Nephrology, University Hospital of Münster Marienhospital Steinfurt, 48565 Steinfurt, Germany; ch.schulte84@googlemail.com; 2 Department of Medicine D, Division of General Internal Medicine, Nephrology and Rheumatology, University Hospital of Münster, 48149 Münster, Germany; Ulrich.Jehn@ukmuenster.de (U.J.); Katharina.schuette-nuetgen@ukmuenster.de (K.S.-N.); Hermann.Pavenstaedt@ukmuenster.de (H.P.); Barbara.Suwelack@ukmuenster.de (B.S.); Stefan.Reuter@ukmuenster.de (S.R.)

**Keywords:** kidney transplantation, tacrolimus, metabolism, C/D ratio, cholesterol, dyslipidemia, LDL-C

## Abstract

Fast tacrolimus (Tac) metabolism is associated with reduced survival rates after renal transplantation (RTx), mainly due to cardiovascular events. Because dyslipidemia is a leading cause of cardiovascular death, we hypothesized that most RTx patients do not achieve recommended target low-density lipoprotein cholesterol (LDL-C) levels (European cardiology society guidelines) and that fast Tac metabolizers have higher dyslipidemia rates. This study included RTx recipients who received initial immunosuppression with immediate-release tacrolimus (IR-Tac), mycophenolate, and prednisolone. Patients were grouped according to their Tac concentration-to-dose ratio (C/D ratio) 3 months after RTx. Dyslipidemia parameters were analyzed at RTx, 3 months, and 12 months after RTx. Statin use and renal function were documented in a 12-month follow-up, and death was documented in a 60-month follow-up. Ninety-six RTx recipients were divided into two groups: 31 fast Tac metabolizers (C/D ratio < 1.05 ng/mL·1/mg) and 65 slow metabolizers (C/D ratio ≥ 1.05 ng/mL·1/mg). There were no differences in triglyceride or cholesterol levels between groups at RTx, 3, and 12 months after RTx. A total of 93.5% of fast and 95.4% of slow metabolizers did not achieve target LDL-C levels (*p* = 0.657). Fast metabolizers developed lower renal function compared to slow metabolizers 12 months after RTx (*p* = 0.009). Fast metabolizers showed a 60 month survival rate of 96.8% compared to 94.7% in the slow metabolizer group (*p* = 0.811). As most RTx recipients do not reach recommended target LDL-C levels, individualized nutritional counseling and lipid-lowering therapy must be intensified. Fast Tac metabolism is associated with lower renal function after RTx, but does not play a significant role in dyslipidemia.

## 1. Introduction

Renal transplantation (RTx) is the preferred renal replacement procedure compared to dialysis [1]. Nevertheless, many RTx recipients are considered high-risk patients for cardiovascular (CV) events [2]. Firstly, most RTx recipients do not reach estimated glomerular filtration rates (eGFRs) compared with healthy controls, which is important because the eGFR has an inverse relationship with cardiovascular disease [3,4]. Secondly, immunosuppression with corticosteroids or calcineurin inhibitors (CNIs) is often associated with the development of dyslipidemia. Thirdly, CV risk associated with cholesterol levels tended to be higher in transplant recipients than in the Framingham Heart Study population in [5]. Recently, the European Society of Cardiology (ESC) published target low-density lipoprotein cholesterol (LDL-C) levels for different CV risk groups [2]. Individuals at moderate CV risk are recommended to achieve LDL-C levels < 100 mg/dL, high-risk patients < 70 mg/mL, and very-high-risk patients < 55 mg/dL. Therefore, most RTx recipients should achieve a target LDL-C level of at least < 70 mg/mL. Recently, the CKD-REIN study collaborators showed that patients with chronic kidney disease (CKD) in stages G3a–5 who are eligible for lipid-lowering therapy are frequently untreated, and those who receive therapy rarely achieve LDL-C targets [6].

Several studies showed an association between fast tacrolimus (Tac) metabolism and increased mortality [7,8]. As described for CKD patients [3], CV events were the main reason of death in these cohorts. Therefore, we initially hypothesized that most RTx recipients do not achieve recommended LDL-C levels as suggested by the ESC [9]. It was also shown that fast Tac metabolism is associated with an increased decline of renal function compared with slow metabolizers [7,10,11], but there is currently no data on Tac metabolism and dyslipidemia. Accordingly, we hypothesized that fast Tac metabolism is related to higher triglyceride and cholesterol levels, which may promote CV disease.

## 2. Patients and Methods

### 2.1. Patients and Study Design

**Figure 1:**
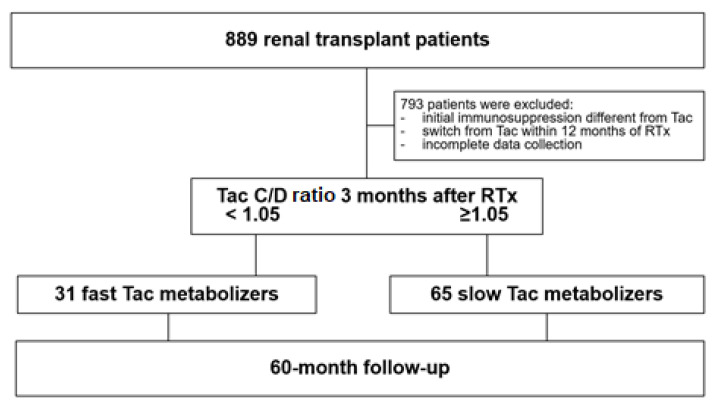
Study design and patient enrollment. Initially, a total of 889 renal transplant recipients were screened, but 793 patients were excluded because they did not meet the inclusion criteria. RTx recipients were defined as fast and slow Tac metabolizers 3 months after transplantation, and survival was observed in a 60-month follow-up. Abbreviations: Tac, tacrolimus; RTx, renal transplantation. C/D ratio values in ng/mL·1/mg.

This retrospective, observational study was performed considering patients who had undergone RTx at the University Hospital of Münster from 2007 to 2015. Figure 1 illustrates the enrollment of the subjects in the study. The study included 96 patients who met the inclusion criteria: age ≥ 18 years, intake of immediate-release Tac (IR-Tac) since RTx and available lipid status at RTx, 3, and 12 months after. The initial immunosuppressive regimen consisted of basiliximab, tacrolimus (target trough 6–12 ng/mL for 3 months, thereafter 4–8 ng/mL), mycophenolate mofetil, and prednisolone that was reduced to a maintenance dosage of 5 mg once daily (q.d.) at 3–6 months. Patient data were collected from the hospital’s electronic health records. Blood analyses were performed using a Roche modular platform (Cobas, Roche Diagnostics, Mannheim, Germany), and renal function (based on enzymatic creatinine measures) was determined by calculating the eGFR using the CKD-EPI (Chronic Kidney Disease Epidemiology Collaboration) equation.

**Figure 2:**
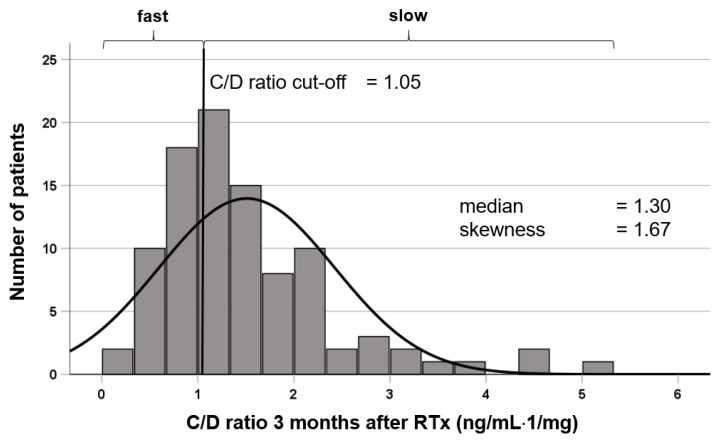
Histogram of the distribution of the tacrolimus C/D ratio (ng/mL·1/mg). The study cohort showed an asymmetric distribution relating to their C/D ratios and were categorized in two groups according to previous studies [7,8]. Fast metabolizers had a C/D ratio <1.05 and slow metabolizers ≥1.05 ng/mL·1/mg. RTx, renal transplantation.

The Tac concentration-to-dose ratio (C/D ratio, fast metabolizers C/D ratio < 1.05 ng/mL·1/mg, slow metabolizers C/D ratio ≥ 1.05 ng/mL·1/mg) was calculated to determine the Tac metabolism rate 3 months after RTx, as previously published by us and others [7,8]. The distribution of the C/D ratio in our cohort is shown in Figure 2. The local ethics committee (No. 2014-381-f-N) approved the study. The methods in this study were performed in accordance with the current transplantation guidelines and the Declarations of Istanbul and Helsinki. All participants gave written informed consent for the collection of their clinical data at the time of transplantation. As recommended by the KDIGO (Kidney Disease: Improving Global Outcomes) guideline [12], the lipid profile was determined at months 3 and 12 after transplantation at our center. Depending on the result, we advised the patient (lifestyle management) and recommended statin therapy, which usually consisted of fluvastatin or pravastatin. Nevertheless, the suggested therapeutic approach was usually a recommendation that the patient previously discussed with their treating nephrologist. If the therapeutic goal was not achieved after 12 months, we usually increased the statin dose, supplemented ezetimibe, or recommended presentation to a lipid specialist.

Rates of calcineurin-inhibitor-induced nephrotoxicity (CNIT), BK viral nephropathy (BKVN), and acute rejection (AR) were assessed from indication biopsies. New-onset diabetes after transplantation (NODAT) data were obtained from the medical records, and the need for antidiabetic therapy or diabetes diet was assessed.

### 2.2. Statistical Analysis

SPSS^®^ Statistics 27 for Windows (IBM Corporation, Somers, NY, USA) was used for statistical analyses. Normally distributed data are presented as mean ± standard deviation and non-normally distributed data are shown as median (minimum–maximum). The *t*-test was used for normally distributed data of unrelated groups. Non-normally distributed data were compared with the Mann–Whitney U test, and categorical variables with Fisher’s exact test. Two-sided tests were applied in all statistical evaluations and a *p*-value of ≤0.05 was considered significant for all tests performed.

## 3. Results

The study cohort included 31 fast Tac metabolizers and 65 slow metabolizers. The fast metabolizer group included noticeably more patients with diabetes mellitus at RTx (Table 1).

At the 3 month mark after RTx, fast Tac metabolizers showed noticeably lower Tac trough levels (*p* = 0.004), had received higher Tac doses (*p* > 0.001) but lower prednisolone doses (*p* = 0.015), and had lower C/D ratios (*p* < 0.001) (Table 2). One year after RTx, Tac trough levels and prednisolone doses were comparable between groups, but Tac daily doses were higher in fast metabolizers, resulting in lower C/D ratios (0.96 vs. 1.59 ng/mL·1/mg, *p* < 0.001). Rates of CNIT, BKVN, AR, and NODAT always tended to show worse outcomes in fast metabolizers compared to slow metabolizers.

Statin use was more frequent in fast metabolizers (*p* = 0.024) but—similarly to TC, LDL-C, HDL-C, and triglyceride levels—did not differ between groups at 3 and 12 months after RTx (Table 2). In a subgroup analysis, the lipid profiles between female and male patients and patients <50 and ≥50 years of age in fast and slow metabolizer groups were analyzed. There were no differences between these subgroups (data not shown).

According to current ESC guidelines, most RTx recipients in our cohort were defined as “high-risk” or “very-high-risk” patients [2], with no differences between metabolizer groups (*p* = 0.259, Table 3). Only 6.5% of fast metabolizers and 4.6% of slow metabolizers achieved their individual guideline-compliant LDL-C target value. While 1 out of 4 fast Tac metabolizers in the “moderate-risk” group reached their LDL-C target at all three time points, only 18.5% of slow metabolizers did so. In “high-risk” patients, the current LDL-C target values were rarely reached by patients (6.5% vs. 1.5%) and the “very-high-risk” target values were not reached by any of the patients (Table 3; overall *p* = 0.342). 

**Figure 3:**
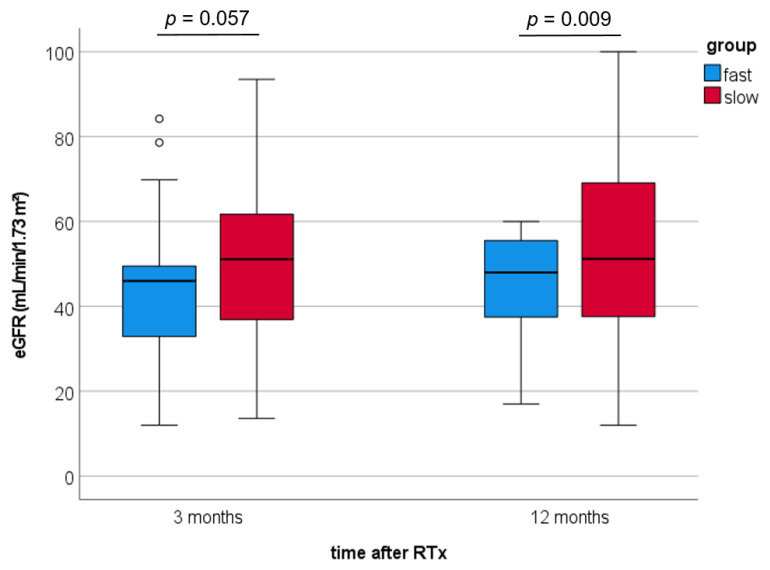
Renal function of fast and slow tacrolimus metabolizers three and twelve months after transplantation. eGFR, estimated glomerular filtration rate; RTx, renal transplantation.

During the follow-up (M12-60), one patient in the fast metabolizer group (3%) and three slow metabolizers (5%) died, mainly from CV events (3 of 4). Three months after RTx, fast metabolizers developed a greater decrease of eGFR by trend (51.1 ± 19.4 vs. 43.7 ± 16.5 mL/min/1.73 m^2^; Figure 3; *p* = 0.057) and a noticeably lower eGFR at 12 months after RTx (53.6 ± 20.9 vs. 44.3 ± 12.9 mL/min/1.73 m^2^; *p* = 0.009) compared to slow metabolizers. 

## 4. Discussion

Herein, we investigated whether Tac metabolism rate is associated with lipid status because we and others had previously observed that mortality was higher in fast metabolizers than in slow metabolizers [7,8]. In addition, it was observed that a lower C/D ratio (<1.8 ng/mL·1/mg) resulted in an increased rate of de novo dyslipidemia one year after liver transplantation [13]. Because CV events are the major cause of death in fast metabolizers and dyslipidemia is clearly associated with mortality in a severity-dependent manner, we conducted the present study [2,14].

The Tac metabolism rate is associated with renal function after transplantation [15]. One year after RTx, fast metabolizers showed lower eGFR values than slow metabolizers. This is in line with recent studies and potentially related to increased rejection rates, BK virus nephropathy, and CNI nephrotoxicity [7,10,16,17,18]. Rates of CNIT, BKVN, and AR always displayed worse outcomes in fast metabolizers as was previously shown in larger cohorts, but the differences did not reach significance in our study cohort, most likely because of small patient numbers [10,17,19]. Renal function after RTx is strongly associated with patient and graft survival [20]. For example, patients with lower eGFR show higher blood pressure values and poorer blood pressure control despite the increased number of antihypertensive medications [21]. However, in this study cohort, the diagnosis of arterial hypertension did not differ between groups. This could be related to the fact that a very large number of patients (>80%) in both groups required blood pressure treatment, which is not unusual because hypertension is common in RTx patients [22].

Interestingly, the rate of diabetes before transplantation was higher in the fast metabolizer group, while the rate of NODAT at three and twelve months after RTx only tended to be higher in the fast metabolizer group (Table 2). This could be important because diabetes influences dyslipidemia [2]. However, diabetic metabolism and dyslipidemia are not always revealed because LDL-C levels may be within the normal range. More typical results are elevated TGs or low HDL-C. Similar findings for dyslipidemia are described in relation to renal function, which was lower in fast metabolizers in our cohort [2]. Interestingly, three and twelve months after RTx, higher TG levels were found more often in fast than in slow metabolizers, whereas the other lipid parameters were relatively similar between groups. Consistent with data from other cohorts of CKD patients who did not achieve target LDL-C levels [6], most RTx recipients in our study also did not achieve their individual goals set by current or previous ESC guidelines [2,23]. 

CNIs and steroids are known to impact lipid metabolism [24]. However, cyclosporine A appears to be less effective than Tac, as it has been shown that switching from cyclosporine A to Tac can improve dyslipidemia [25,26,27]. The reduction in serum LDL-C after switching to Tac may be (partly) caused by removing the inhibitory effect of cyclosporin A on LDL-C receptor production. Interestingly, LDL-C reduction was found only in patients who were not treated with statins [25]. Furthermore, lowering Tac trough levels from 9.5 to 6.4 ng/mL (a Tac level range comparable to that of our patients) did not significantly lower TC, LDL-C, or TG levels in renal transplant recipients, in contrast to steroid withdrawal, which resulted in a slight improvement in lipids [28]. Others found no correlation between Tac trough level, exposure, or Tac dosage and the lipid parameters [29,30]. Unfortunately, we cannot comment on the influence of Tac AUC values in this regard, but we and others have previously shown that Tac AUC is comparable in fast and slow metabolizers [16,31]. The choice of the 3 month time point for the calculation of the C/D ratio was a compromise. We already know from previous analyses that the calculation of the C/D ratio at very early time points (postoperative day 1–10) is not able to predict the metabolism type at all [32]. However, we found acceptable correlations between the 3 month time point and the calculation at 1 month or 6 months [7]. Jouve et al. analyzed the C/D ratio at 3, 6, and 12 months using the same cut-off (1.05 ng/mL·1/mg) and observed no statistically or clinically significant intrapatient evolution of the C/D ratio over time [8]. Rostaing et al. observed that from the third month after transplantation, the C/D ratio was relatively constant over time [33]. The choice of the C/D ratio cut off level can be very relevant [34]. In our first study on the C/D ratio, we assessed the outcomes of three different C/D ratio groups (<1.05, ≥1.05 and <1.54, and ≥1.55 ng/mL·1/mg). Since kidney transplant recipients with a C/D ratio between 1.05 and 1.54 ng/mL·1/mg showed comparable results to patients with a CD ratio ≥1.55 ng/mL·1/mg, we chose to combine both groups in later analyses. However, others chose different C/D ratio cut-off values, which may lead to different definitions and outcomes [18,35].

Consistent with the literature on Tac and lipid metabolism, in the present study we excluded relevant effects of Tac metabolic type on lipid metabolism within 12 months after RTx.

The strengths of the current study included the complete data set of each patient and the use of real-world data that better reflected the reality of treatment. Limitations of this study included (i) the retrospective nature of the study, (ii) the small number of patients, (iii) the lack of adherence data, (iv) the low achievement of therapeutic lipid targets based on current guidelines, and (v) the lack of clear differentiation between effects of renal function, Tac, or co-medication on lipids. However, a prospective multicenter study focusing on the C/D ratio would be desirable to prospectively validate the hypotheses obtained from retrospective studies.

In conclusion, although dyslipidemia after RTx is common (at least at our center), treatment according to current guidelines is suboptimal and individualized nutritional counseling and lipid-lowering therapy must be intensified. The Tac metabolism type does not seem to be a crucial parameter regarding dyslipidemia after RTx. This study is intended to raise awareness of lipid management under real-life conditions and to call for (re-)evaluation of the procedure for individual centers.

## Figures and Tables

**Table 1:** Patient characteristics and immunosuppression.

	Fast Metabolizers (*n* = 31)	Slow Metabolizers (*n* = 65)	*p*-Value
Age (years)	51.9 ± 12.6	51.0 ± 14.8	0.770 ^a^
Sex (m/f)	22 (71%)/9 (29%)	45 (69%)/20 (31%)	1 ^b^
Weight (kg)	79.7 ± 19.2	74.1 ± 14.7	0.158 ^a^
Height (cm)	176 ± 11	172 ± 9	0.108 ^a^
BMI (kg/m^2^)	25.4 ± 4.4	24.7 ± 4.2	0.503 ^a^
Living donor transplantation	10 (32%)	16 (25%)	0.467 ^b^
ESP	4 (13%)	9 (14%)	1 ^b^
Cold ischemic time (h)	8.1 ± 5.8	8.6 ± 4.7	0.698 ^a^
Warm ischemic time (min)	31.6 ± 5.9	32.6 ± 7.5	0.483 ^a^
DGF	2 (7%)	9 (14%)	0.494 ^b^
Time on dialysis	47.5 ± 42.0	59.5 ± 44.6	0.206 ^a^
Previous transplantation	5	5	0.284 ^b^
One previous transplantation	3	5	0.168 ^b^
Two previous transplantations	2	0	
Combined liver transplantation	1	2	1 ^b^
Comorbidities			
Diabetes mellitus	5 (16%)	2 (3%)	0.034 ^b^
Arterial hypertension	25 (81%)	59 (91%)	0.193 ^b^
BMI ≥ 25 kg/m^2^	18 (58%)	36 (55%)	0.830 ^b^
Donor Characteristics			
Donor age	51.0 ± 15.3	53.4 ± 14.5	0.475 ^a^
Donor sex (m/f)	10 (32%)/21 (68%)	30 (46%)/35 (54%)	0.269 ^b^

BMI, body mass index; ESP, European Senior Program; DGF, delayed graft function. *p*-Values: ^a^ Welch’s *t*-test; ^b^ Fisher’s exact test.

**Table 2:** Immunosuppression, statins, complications, cholesterol levels, and triglycerides.

	Fast Metabolizers (*n* = 31)	Slow Metabolizers (*n* = 65)	*p*-Values
Tac trough level M3 (ng/mL)	6.8 ± 2.4	8.4 ± 2.8	0.004 ^a^
Tac daily dose M3 (mg)	9.0 (3–20)	5 (2–12)	<0.001 ^b^
Tac C/D ratio M3 (ng/mL/mg)	0.79 (0.28–1.00)	1.57 (1.05–5.15)	<0.001 ^b^
Tac daily dose M12 (mg)	7 (2–18)	3.75 (1–11)	<0.001 ^b^
Tac trough level M12 (ng/mL)	6.8 ± 2.1	6.3 ± 2.1	0.401 ^a^
Tac C/D ratio M12 (ng/mL/mg)	0.96 (0.28–2.85)	1.59 (0.24–7.60)	<0.001 ^b^
Prednisolone M3 (mg)	7.5 (0–50)	10 (5–30)	0.015 ^b^
Prednisolone M12 (mg)	5 (0–20)	5 (3–20)	0.594 ^b^
Statin at discharge	8 (26%)	5 (8%)	0.024 ^c^
Statin M3	10 (32%)	18 (28%)	0.640 ^c^
Statin M12	18 (58%)	39 (60%)	1 ^c^
**Complications**			
CNIT until M12	4 (12.9%)	4 (6.2%)	0.268 ^c^
BKVN until M12	3 (9.7%)	1 (1.5%)	0.097 ^c^
AR until M12	6 (19.4%)	6 (9.2%)	0.193 ^c^
NODAT until M3	4 (12.9%)	7 (10.8%)	0.743 ^c^
NODAT between M3 and M12	5 (16.1%)	10 (15.4%)	1 ^c^
**TC**			
At RTx	195 (85–455)	200 (119–395)	0.422 ^b^
3 months	215 (119–284)	224 (125–353)	0.285 ^b^
12 months	209 (109–353)	202 (121–378)	0.443 ^b^
**LDL-C**			
At RTx	102 (11–372)	111 (39–274)	0.565 ^b^
3 months	117 (57–194)	122 (56–229)	0.347 ^b^
12 months	116 (43–269)	114 (47–266)	0.919 ^b^
**HDL-C**			
At RTx	45 (25–87)	48 (22–119)	0.464 ^b^
3 months	49 (31–91)	52 (28–91)	0.426 ^b^
12 months	46 (31–108)	51 (24–104)	0.148 ^b^
**TG**			
At RTx	158 (57–469)	159 (67–885)	0.763 ^b^
3 months	221 (90–545)	186 (46–1326)	0.283 ^b^
12 months	206 (68–774)	160 (77–663)	0.138 ^b^

Tac, tacrolimus; M, month; C/D ratio, concentration/dose ratio; CNIT, calcineurin inhibitor-induced nephrotoxicity; BKVN, BK virus nephropathy; AR, acute rejection; NODAT, new-onset diabetes after transplantation; RTx, renal transplantation; TC, total cholesterol; LDL-C, low-density lipoprotein cholesterol; HDL-C, high-density lipoprotein cholesterol; TG, triglycerides. *p*-Values: ^a^ Welch’s *t*-test; ^b^ Mann–Whitney U test; ^c^ Fisher’s exact test.

**Table 3:** Cardiovascular risk and achieved LDL-C levels.

	Fast Metabolizers (*n* = 31)	Slow Metabolizers (*n* = 65)	*p*-Values
**CV risk level according to the ESC guidelines**			
Moderate-risk	4 (12.9%)	7 (10.8%)	0.259
High-risk	17 (54.8%)	34 (52.3%)	
Very-high-risk	10 (32.3%)	14 (21.5%)	
LDL-C target level achieved *			
Individual level achieved	2 (6.5%)	3 (4.6%)	0.657
Moderate-risk achieved	8 (25.8%)	12 (18.5%)	0.342
High-risk level achieved	2 (6.5%)	1 (1.5%)	
Very-high-risk level achieved	0	0	

CV, cardiovascular risk; LDL-C, low-density lipoprotein cholesterol. *p*-Values: Fisher’s exact test. * According to current European Society of Cardiology guidelines 2019.
